# Comparative Transcriptome Analysis Revealing the Different Germination Process in Aryloxyphenoxypropionate-Resistant and APP-Susceptible Asia Minor Bluegrass (*Polypogon fugax*)

**DOI:** 10.3390/plants9091191

**Published:** 2020-09-12

**Authors:** Xiaoyue Yu, Wei Tang, Yongjie Yang, Jianping Zhang, Yongliang Lu

**Affiliations:** State Key Laboratory of Rice Biology, China National Rice Research Institute, Hangzhou 311400, China; yuxiaoyue@caas.cn (X.Y.); tangwei@caas.cn (W.T.); yangyongjie@caas.cn (Y.Y.); zhangjieping@caas.cn (J.Z.)

**Keywords:** fitness cost, carbohydrate metabolism, fatty acid metabolism, hormone regulation

## Abstract

Herbicide-resistant mutations are predicted to exhibit fitness cost under herbicide-free conditions. Asia minor bluegrass (*Polypogon fugax*) is a common weed species in the winter crops. Our previous study established a *P. fugax* accession (LR) resistant to aryloxyphenoxypropionate (APP) herbicides, which also exhibited germination delay relative to the susceptible accession (LS). A comparative transcriptome was conducted to analyze the gene expression profile of LS and LR at two germination time points. A total of 11,856 and 23,123 differentially expressed genes (DEGs) were respectively identified in LS and LR. Most DEGs were involved in lipid metabolism, carbohydrate metabolism, amino acid metabolism, and secondary metabolites biosynthesis. Twenty-four genes involved in carbohydrate and fatty acid metabolism had higher relative expression levels in LS than LR during germination. Nine genes involved in gibberellin (GA) and abscisic acid (ABA) signal transduction showed different expression patterns in LS and LR, consistent with their different sensitivity to exogenous hormones treatments. This study first provided insight into transcriptional changes and interaction in the seed germination process of *P. fugax*. It compared the differential expression profile between APP herbicides resistance and susceptible accessions during germination, which contributed to understanding the association between herbicide resistance and fitness cost.

## 1. Introduction

Herbicide resistant mutations are considered as pre-existed or arise spontaneously at a very low rate within weeds populations [1,2,3]. With frequent herbicide application, herbicide-resistant mutations are rapidly selected and enriched in weed populations [4]. Without herbicide stress, herbicide-resistant mutations are predicted to exhibit an adaptation cost (fitness cost) due to the impaired enzyme ability or altered feedback inhibition resulting in insufficient or excessive product biosynthesis [5,6,7]. Many herbicide-resistant weeds exhibited altered phenotypes, including seed germination ability, growth cycle, biomass production, competitive ability, and reproduction [8,9]. For instance, the acetolactate synthase (ALS) and acetyl-CoA carboxylase (ACCase) herbicides multiple-resistance *Lolium rigidum* biotypes showed lower vigor and competitive ability against wheat than that of the susceptible biotype [10]. The ACCase mutant alleles in *Alopecurus myosuroides* showed a reduction of biomass, height, and seed production [11]. Two ACCase mutant alleles exhibited lower growth rate, biomass, and seed production in *Hordeum glaucum* [12].

Asia minor bluegrass (*Polypogon fugax*) is a common winter weed species distributed across China and other Asian countries. This annual grass has an extended period of emergence from early November to late December. Its life cycle is highly close to several winter crops, including wheat (*Triticum aestivum* L.), rapeseed (*Brassica napus* L.), and some vegetables [13,14]. *Polypogon fugax* is a competitive weed, especially in moist soil, and has become an increasing problem in wheat or rapeseed fields in rotation with rice [15]. In our previous study, a *P. fugax* accession (LR) collected from Sichuan Province in China was highly resistant to aryloxyphenoxypropionate (APP) herbicides due to an Ile-2041-Asn substitution in ACCase protein [16]. Besides, this resistance accession also exhibited fitness cost, including lower germination and emergence potential, earlier flowering, and tiller and panicle emergence and seed shedding, compared with the susceptible accession [17,18]. A transcriptome study of the *P. fugax* resistant and susceptible accessions were performed at the flowering stage, which provided a genomic resource for understanding the molecular basis of early flowering. The study identified twelve genes with different expression pattern in herbicide resistant and susceptible plants [19]. However, the association between germination delay and herbicide resistance is still unclear. The fitness cost occurred in herbicide resistant mutations are not universal, which depends on the particular mutation [20,21,22], genetic background [23], and environmental conditions [24,25]. Thus, the susceptible accession in this study was collected from the same habitat of resistant accession to minimize the genetic background variance. The resistant and susceptible accessions reproduced three generations under the same environmental condition to minimize the influence of environmental conditions. These limitations allowed us to focus on the effect of herbicide resistance on fitness cost.

Seed germination and dormancy have a complex regulation network, including energy production, protein metabolism, phytohormone control, and transcription factors regulation. With the development of genomics, transcriptomic analysis is one of the most effective and economic approaches to investigate the difference of germination process between the tested samples. The aim of this study is to investigate the expression profile of the seed germination process in the APP herbicides-resistant and susceptible *P. fugax* accessions and try to figure out the relevant biological pathways or function genes that lead to germination delay.

## 2. Results

### 2.1. Characterization of Seed Germination in LS and LR

**Figure 1:**
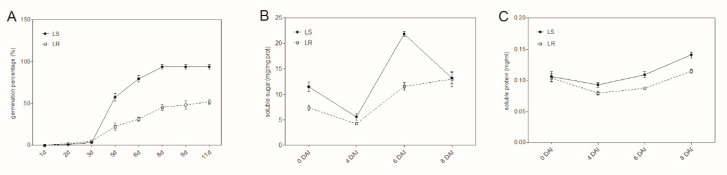
Seed germination characters in a *Polypogon fugax* accession susceptible to aryloxyphenoxypropionate (APP) herbicides (LS and a *P. fugax* accession resistant to APP herbicides (LS). (**A**) Dynamic change of germination percentage of LS and LR seeds; dynamics change of soluble sugar (**B**) and soluble protein content (**C**) of LS and LR seeds. The solid line represents LS and the dotted line represents LR. DAI means days after incubated. Bars are mean ± standard error (n = 5).

The maximum germination (gMAX) of LR was about 52%, while that of LS reached 94% (Figure 1A), consistent with the previous study, suggesting that the low germination character was steadily inherited in LR. According to the germination curve, the rapid growth period was from 3 days after incubated (DAI) to 8 DAI. The content of soluble sugar in the 0 DAI seeds of LS was higher than LR. After incubation, the soluble sugar content deceased and then dramatically increased at 6 DAI in LS and LR seeds. The soluble sugar content of LR seeds was always lower than that of LS seeds until 8 DAI (Figure 1B). Similarly, the content of soluble protein decreased first and then increased. The content of LR seeds was lower than that of LS during the germination process (Figure 1C). Combined with the dynamic change of germination rate and soluble sugar and protein content, we chose 3-DAI and 6-DAI seeds for transcriptome analysis to compare the difference of germination process between LS and LR.

### 2.2. De Novo Assembly of P. fugax Reference Transcriptome

**Figure 2:**
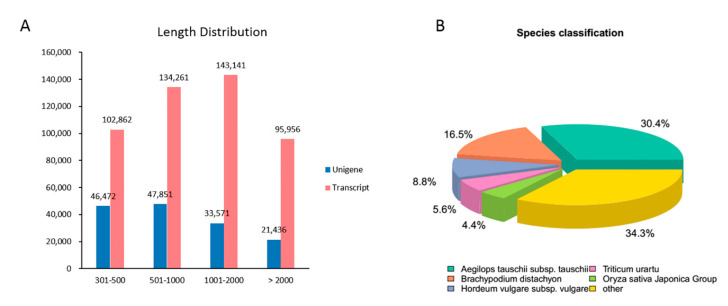
Transcriptome assembly and unigenes annotation of *P. fugax*. (**A**) The length distribution of unigenes and transcripts; (**B**) species classification of *P. fugax* transcriptome annotated to NR (NCBI non-redundant protein sequences) database. The numbers near the pie charts indicate the percentage of unigenes in each class.

A total of 12 samples were used to conduct the RNA-seq analysis. The number of total clean reads of each sample was shown in Supplemental Table S1. A total of 476,220 transcripts and 149,330 genes were de novo assembled, and the distribution of length interval was shown in Figure 2A. The number of annotated unigenes of each database was show in Supplemental Table S2. Basically, a total of 99,209 genes (66.43%) were at least annotated to one database, suggesting that de novo transcriptome had relatively complete gene function information. Among them, 67,183 genes (44.98%) were annotated to NR database, and established that *P.fugax* was highly similar to *Aegilops tauschii* subsp. *tauschii* (30.4%), *Brachypodium distachyon* (L.) P. *Beauv*. (16.5%), *Hordeum vulgare* subsp. *vulgare* (8.8%), *Triticum urartu* (5.6%), and *Oryza sativa* Japonica Group (4.4%) (Figure 2B).

### 2.3. Identification of Differentially Expressed Genes (DEGs)

**Figure 3:**
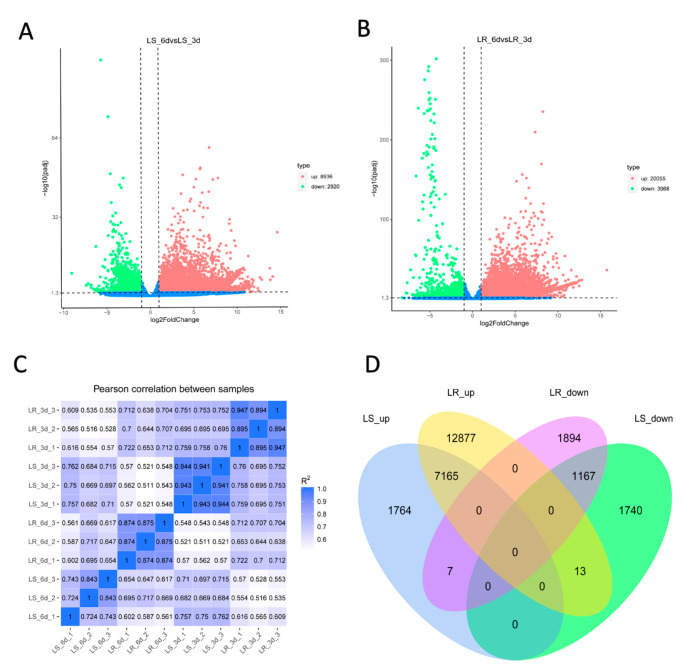
Analysis of differentially expressed genes (DEGs) in LS comparison setting (LS_6d vs. LS_3d) and LR comparison setting (LR_6d vs. LR_3d). The volcano plot of DEGs in LS (**A**) and LR (**B**). Green dots represent the significantly downregulated DEGs. Red dots represent the significantly upregulated DEGs (padj < 0.05). (**C**) Correlation cluster between three biological replicates of LS_3d, LS_6d, LR_3d, and LR_6d. (**D**) Venn diagram of upregulated and downregulated DEGs in LS and LR comparison settings. LS_up: upregulated DEGs in LS; LR_up: upregulated DEGs in LR; LS_down: downregulated DEGs in LS; LR_down: downregulated DEGs in LR.

To determine the molecular basis involved in the germination process of LS and LR, two comparison settings (LS_6d vs. LS_3d and LR_6d vs. LR_3d) were analyzed. The biological samples had good reproducibility due to the high correlation values (Figure 3C). The distribution of the DEGs in LS and LR were directly exhibited in volcano plots (Figure 3A,B). A total of 11,856 DEGs were identified; of them, 8936 were upregulated and 2920 were downregulated in LS comparison. A total of 23,123 DEGs were identified; of them, 20,055 were upregulated and 3068 were downregulated in LR comparison. A venn diagram showed that 7165 DEGs were upregulated and 1167 DEGs were downregulated in both LS and LR comparison settings (Figure 3D). Meanwhile, seven DEGs were upregulated in LS but downregulated in LR comparison, and 13 DEGs were downregulated in LS but upregulated in LR comparison. The majority of them were annotated as uncharacterized or hypothetical proteins (5/7 and 8/13). Two DEGs involved in ABA biosynthesis and signal pathways were downregulated in LS but upregulated in LR, indicating that the ABA regulation network might be different in LS and LR during germination (Supplemental Table S3).

### 2.4. GO (Gene Ontology) and KEGG (Kyoto Encyclopedia of Genes and Genomes) Enrichment of DEGs

To understand the difference in biological functions and processes related to germination between LS and LR, all the DEGs were enriched to GO and KEGG database. The upregulated DEGs in LS were enriched to 109 GO terms, and the downregulated DEGs were enriched to 9 GO terms (Supplemental Table S4). The upregulated DEGs in LR were enriched to 224 GO terms and the downregulated DEGs were enriched to 40 GO terms (Supplemental Table S5). The top enriched GO terms of the upregulated DEGs in LS and LR were similar, such as “metabolic process,” “catalytic activity,” and “single-organism process.” With regard to the downregulated DEGs, the enriched GO terms were different and much less in LS than LR.

**Figure 4:**
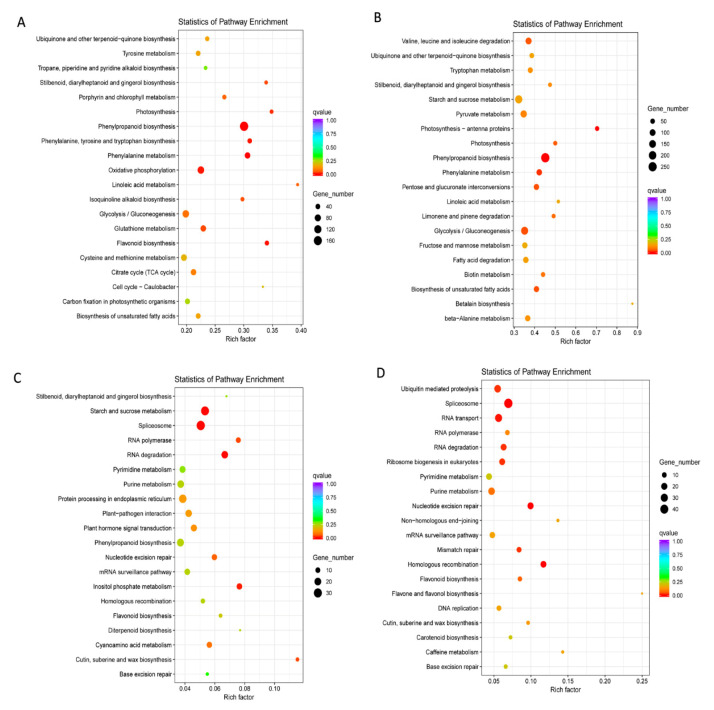
KEGG enrichment analysis of the upregulated DEGs. (**A**) Top 20 enriched pathways of upregulated DEGs in LS comparison setting. (**B**) Top 20 enriched pathways of upregulated DEGs in LR comparison setting. (**C**) Top 20 enriched pathways of downregulated DEGs in LS comparison setting. (**D**) Top 20 enriched pathways of downregulated DEGs in LR comparison setting. The colors are shaded according to the q-values level as shown in the color bars; the size of the circle indicates the number of DEGs.

Likewise, all the DEGs in LS and LR were enriched to KEGG database. The top 20 enriched pathways of upregulated DEGs in LS and LR were shown in Figure 4A,B; of them, eight pathways were same in LS and LR. The majority of upregulated DEGs were related to lipid metabolism, carbohydrate metabolism, amino acid metabolism and secondary metabolites biosynthesis in LS and LR. The top 20 enriched KEGG pathways of downregulated DEGs in LS and LR were shown in Figure 4C,D; of them, 11 pathways were the same in LS and LR. Some of the pathways were related to the genetic process, such as spliceosome, RNA degradation, and nucleotide excision repair, which were not found in the upregulated DEGs enrichment. Starch and sucrose metabolism was the only pathway in the LR upregulated DEGs enrichment and the LS downregulated DEGs enrichment. According the biological functions of DEGs and the relevant processes of germination, we selected some pathways to investigate the different regulation networks in LS and LR (Table 1).

### 2.5. DEGs Related to Fatty Acid Metabolism

ACCase is the rate-limiting enzyme in fatty acid metabolism. Six DEGs were significantly different expressed in LS and LR during germination, including gene (Cluster-39490.0) coding ACCase biotin carboxylase (ACACA), gene (Cluster-31226.0) coding 3-oxoacyl-[acyl-carrier protein] reductase (FabG), gene (Cluster-37472.4287) coding long-chain acyl-CoA synthetase (ACSL), gene (Cluster-33548.0) coding acetyl-CoA acyltransferase 1 (ACAA1), gene (Cluster-37472.85335) coding acyl-CoA dehydrogenase (ACADM), gene (Cluster-30044.0) coding acetyl-CoA C-acetyltransferase (ACAT). All of them were upregulated in LS and LR from 6 DAI to 3 DAI, but the increasing level were much lower in LR than LS. The results suggested that these genes involved in fatty acid metabolism were more actively expressed in LS during germination.

### 2.6. DEGs Related to Carbohydrate Metabolism

One of the major changes during germination is a rapid increase in respiration, which involves glycolysis, oxidative pentose phosphate pathway, TCA cycle, and oxidative phosphorylation. Six DEGs involved in glycolysis were upregulated in LS and LR with higher upregulation level in LS, including gene (Cluster-37472.6545) coding glucose-6-phosphate isomerase (GPI), gene (Cluster-37472.71503) coding fructose-bisphosphate aldolase (FBA), gene (Cluster-35171.2) coding phosphoenolpyruvate carboxykinase (PCKA), gene (Cluster-40718.0) coding pyruvate dehydrogenase E1 component alpha subunit (PDHA), and genes (Cluster-37472.39187, Cluster-37472.47005) coding alcohol dehydrogenase (ADH).

Seven DEGs involved in TCA cycle had higher upregulation in LS than LR, including genes (Cluster-37472.85108, Cluster-15133.3) coding malate dehydrogenase (MDH), gene (Cluster-37472.21027) coding citrate synthase (CS), gene (Cluster-33485.0) coding 2-oxoglutarate dehydrogenase E1 component (OGDH), genes (Cluster-38121.0, Cluster-34010.0) coding succinyl-CoA synthetase alpha subunit and beta subunit (LSC1 and LSC2), genes (Cluster-37472.82495, Cluster-39676.0) coding succinate dehydrogenase (ubiquinone) iron-sulfur subunit (SDHB), and gene (Cluster-41938.0) coding pyruvate carboxylase (PYC).

Three genes involved in oxidative pentose phosphate pathway showed higher upregulation in LS than LR, including gene (Cluster-37472.72264) coding 6-phosphogluconate dehydrogenase (PGD), gene (Cluster-37472.83492) coding transketolase (TKT) and gene (Cluster-37472.237) coding gluconokinase (gntK).

### 2.7. DEGs Related to Hormones Biosynthesis and Signal Transduction

Seeds germination and dormancy processes are regulated by hormone signal transduction. Three DEGs involved in GA biosynthesis were upregulated in LS and LR from 3 DAI to 6 DAT, and the upregulation level was higher in LS than LR, including genes (Cluster-37472.24032 and Cluster-37472.23672) coding two ent-kaurene synthases (KS) and gene (Cluster-37472.70387) encoding gibberellin 3beta-hydroxylase (GA3ox). The gene (Cluster-51396.0) encoding phytochrome-interacting factor 4 (PIF4), which negatively regulated GA signal transduction, was downregulated in LS but upregulated in LR during germination.

The DEGs involved in ABA biosynthesis showed different regulation level in LR and LS. The gene (Cluster-37472.23110) coding 9-cis-epoxycarotenoid dioxygenase (NCED) was downregulated in LS but not changed in LR during germination. The expression of gene (Cluster-37472.23992) coding phytoene synthase (PSY) and gene (Cluster-37472.76697) coding lycopene epsilon-cyclase (LcyE) were both elevated in LS and LR, and the upregulation level was higher in LR than LS. DEGs related to ABA signal transduction were significantly upregulated in LR but not changed in LS, such as gene (Cluster-37472.6593) encoding serine/threonine-protein kinase SRK2 (SNRK2) and gene (Cluster-48355.0) coding ABA responsive element binding factor (ABF).

### 2.8. Hormones Regulate the Seed Germination of LS and LR

**Figure 5:**
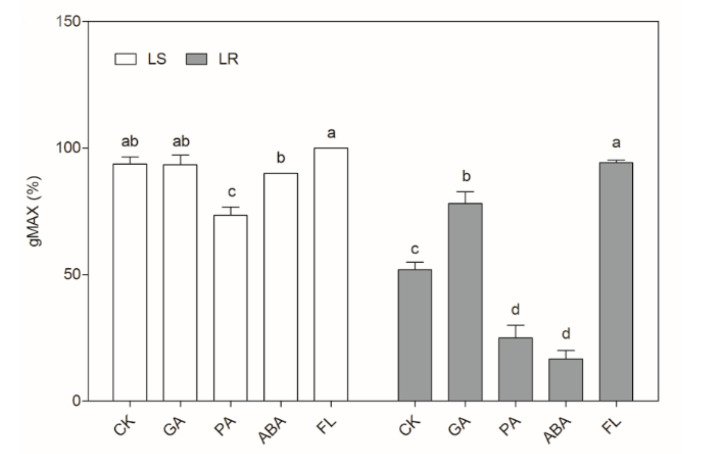
The maximum germination (gMAX) of LS and LR with exogenous hormones application. CK: water, GA: gibberellin, PA: paclobutrazol, ABA: abscisic acid, FL: fluridone. Bars are mean ± standard error (n = 5). Values in a column followed by the different letters means significant difference (*p* ≤ 0.05).

According to the results of RNA-seq, GA and ABA signaling pathways mediated the germination ability of LS and LR. The seeds of LS and LR were treated with exogenous GA and ABA and their synthesis inhibitors, paclobutrazol (PA) and fluridone (FL), to observe the gMAX values (Figure 5). The application of GA significantly elevated the gMAX of LR from 52% to 78%, suggesting that exogenous GA could promote the germination of LR. The application of PA decreased the gMAX of LS to 73%, and that of LR decreased to 25%, suggesting that GA biosynthesis in LR was more strongly inhibited by PA. The application of ABA had no significant effect on the gMAX of LS but decreased that of LR to 17%, suggesting that the inhibitory by ABA was stronger in LR than LS. The application of FL dramatically increased the gMAX of LR to 94%, suggesting that the dormancy of LR was completely removed when ABA biosynthesis was blocked.

### 2.9. Validation of DEGs by Quantitative Real-Time PCR (qRT-PCR)

**Figure 6:**
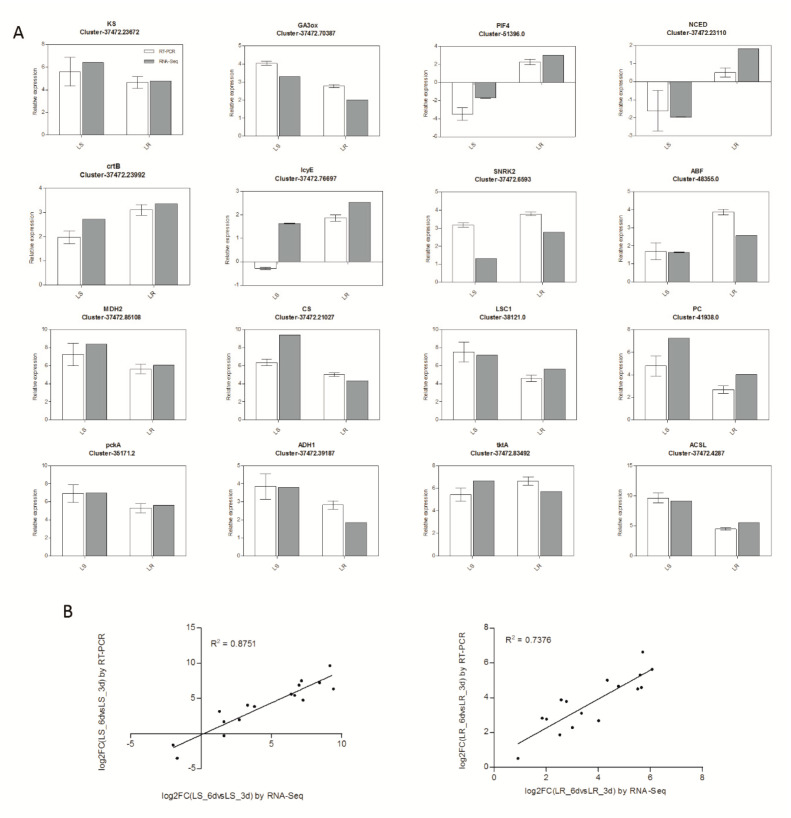
The validation of RNA-seq results using qRT-PCR. (**A**) The relative expression levels of 16 DEGs determine by qRT-PCR (white column) and RNA-Seq (grey column). RNA-Seq results (fold change) and qRT-PCR results (2^−ΔΔCt^) were transformed to log_2_ forms. Bars are mean ± standard error (n = 3). (**B**) Linear regression analysis of the RNA-Seq and qRT-PCR results. The R^2^ values represent the correlation between RNA-Seq and qRT-PCR results.

A total of 16 DEGs were randomly selected to verify the accuracy and reproducibility of the transcriptome results by qRT-PCR (Figure 6). The correlation between transcriptome results (FC) and qRT-PCR results (2^−ΔΔCt^) was calculated using log2 fold variation measurements to produce a scatter plot. The results showed that the expression profiles of these DEGs were consistent with the transcriptome results, with relative R^2^ = 0.8751 and R^2^ = 0.7376 in LS and LR, respectively.

## 3. Discussion

Seed germination is a process with complex physiological, biochemical, and molecular biological bases [26]. In this study, we compared the expression profiles between LS and LR and obtained abundant DEGs in two comparisons. Unexpectedly, the number of upregulated DEGs in LR was much more than LS (20,055 in LR and 8936 in LS). We compared the gene expression profiles between LR and LS at the same time points (LR_3d vs. LS_3d, LR_6d vs. LS_6d). The results showed that most DEGs were downregulated in LR compared with LS, especially at 3 DAI (Supplemental Table S6). This indicated that the gene regulations were lower in LR than LS, corresponding to the lower metabolic activity as described in Figure 1. Many gene expressions exhibited a rise at first and then decreased during the germination process. It is possible that the expression of genes in LS have already peaked and are in the downtrend at 6 DAI, while the expression in LR is still in the uptrend. This might explain why more genes were upregulated in LR from 6 DAI to 3 DAI. In addition, the upregulated DEGs in LR were significantly enriched to starch and sucrose metabolism. However, the downregulated DEGs in LS were enriched to the same pathway. With the absorption of water, starch and sucrose are decomposed into small and soluble molecules that can be easily utilized and transformed to support germination [27]. We assumed that the transduction of starch and sucrose to glucose happened earlier in LS than LR during germination, which is consistent with the previous results that the genes induction were earlier in LS than LR.

**Figure 7:**
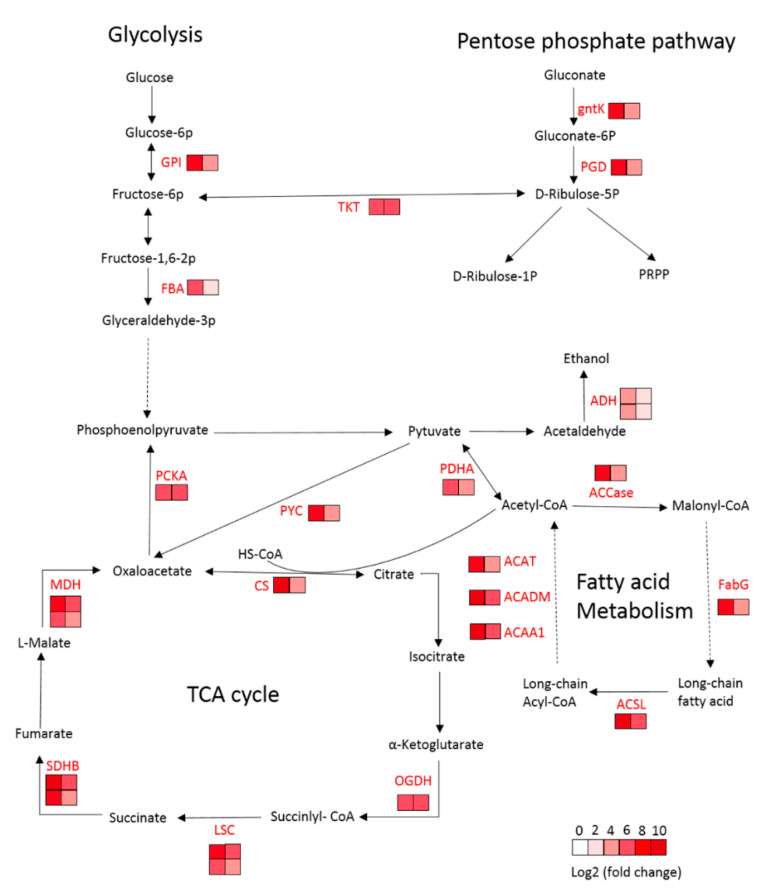
Visualization of DEGs involved in carbohydrate metabolism and fatty acid metabolism. Substrates are in black font, and enzymes in red font. The color of boxes represents Log_2_ (fold change) values based on the transcriptome results. The values are shown by a color gradient from low (white) to high (red). The left column of boxes represents the Log_2_ (fold change) value obtained from LS comparison setting, and the right column represents that from LR comparison setting. Each row of boxes represents one DEG. Solid arrows represent direct processes, and dashed arrows represent indirect processes.

The lipid metabolism, carbohydrate metabolism, or amino acid metabolism play important roles in regulating seed germination and dormancy. APP herbicides inhibit weed growth by interrupting the biological function of the ACCase enzyme, which catalyzes the carboxylation of acetyl-CoA to malonyl-CoA and acts as the initial step of fatty acid biosynthesis [28,29,30]. The APP-herbicides-resistant *P. fugax* accession LR exhibited lower germination in comparison with the susceptible accession LS. Similarly, some ACCase-inhibiting herbicides–resistant weeds also exhibited germination delay and/or dormancy in dark condition [31,32]. It is predictable that the mutations of the ACCase enzyme may change its configuration and geometry and alter normal plant metabolism, leading to a whole plant fitness cost [33]. ACCase has three subunits: biotin carboxylase carrier protein, biotin carboxylase, and carboxyltransferase domains [34]. Molecular and biochemical studies have proved that the carboxyltransferase domain is the target site of APP herbicides [35]. Unfortunately, the transcriptome data did not identify any DEGs annotated as ACCase carboxyltransferase domain in LS or LR. However, the gene (Cluster-39490.0) coding ACCase biotin carboxylase was identified with higher upregulation in LS than LR. Because ACCase is the rate-limiting enzyme in fatty acid metabolism, the upregulation of *ACCase* gene may influence the downstream reactions. Two DEGs involved in fatty acid elongation and termination and three DEGs involved in fatty acid degradation exhibited higher upregulation level in LS in comparison with LR (Figure 7). These results indicated that the fatty acid metabolism was more active in LS than LR during seed germination. In addition, acetyl-CoA, which acts as the precursor of fatty acid biosynthesis, is the product of glycolysis and TCA. DEGs involved in TCA, glycolysis, or the pentose phosphate pathway showed higher upregulation level in LS than LR during germination (Figure 7). The dynamic change of soluble sugar and protein content also confirmed that more soluble sugar and protein participated in carbon oxidation and enzyme catalysis to supply energy in LS compared to LR during germination. Many studies proved that genes in carbohydrate metabolism had important roles in regulating seed germination. For example, *PCKA*-deficient mutants of *Arabidopsis* and tomato showed growth suppression of germinated seedlings [36]. The *MDH* gene mutants in *Arabidopsis* showed slow germination rates, higher content of free amino acids, different sugar levels, and lower content of 2-oxoglutarate [37]. The seeds of the *CS*-deficient *Arabidopsis* were dormant and did not metabolize triacyglycerol [38]. Therefore, the mutation of the *ACCase* gene in LR might cause a chain effect on fatty acid and carbohydrate metabolisms, resulting in less energy being supplied to promote germination.

Besides DEGs involved in carbohydrate and fatty acid metabolism, some genes related to hormone biosynthesis and signal transduction pathways were identified in LS and LR comparison settings. Seeds germination and dormancy processes are regulated by diverse hormones. GA and ABA antagonistically regulate the transition of germination and dormancy, in that GA promotes germination and ABA promotes dormancy [39]. The first step in the GA biosynthetic pathway is transformed geranylgeranyl pyrophosphate to ent-kaurene catalyzed by ent-kaurene synthetases (KS). The *KS*-deficient mutants in *Arabidopsis* showed strong seed dormancy and recovered germinate with exogenous GA treatment [40,41]. GA3ox catalyzes the final biosynthetic step to produce bioactive GAs. Two *Arabidopsis* genes, *GA4* and *GA4H*, encoding GA3ox were highly expressed during seed germination [42]. In this study, two DEGs annotated as KS and one annotated as GA3ox showed higher expression in LS than LR, indicating that GA biosynthesis was more active in LS than LR. Consistent with this result, the inhibitory by PA was more effective in LR than LS. Phytochrome-interacting factor 4 (PIF4) regulates the gibberellin-signaling pathway via DELLA proteins, which blocks the GA signal pathway and results in germination delay [43]. DEG annotated as PIF4 was downregulated in LS but upregulated in LR, indicating that PIF4 might repress seed germination via inhibiting the GA signal transduction in LR. DEGs encoding the proteins involved in ABA biosynthesis and signal transduction pathways showed higher upregulation level in LR than LS during seed germination process. NCED is the key regulatory step of ABA biosynthesis. The *Arabidopsis* mutants of *NCED* genes, *Atnced6* and *Atnced9*, reduced ABA content level in seeds [44]. PSY is the limiting step of carotenoids synthesis, the upstream of ABA biosynthesis. The *PSY* gene overexpressed *Arabidopsis* mutant exhibited delayed germination, and the degree of delay was positively associated with the increased levels of carotenoids and ABA [45]. SNRK2 is activated by ABA and phosphorylate ABF, which is important for the activation of ABA signal transduction [46,47]. DEGs identified as these genes all showed lower regulation level in LS than LR, indicating that the ABA biosynthesis and transduction was less active in LS than LR. Consistent with this, LR was more sensitive to ABA compared with LS and FL could restore the germination ability of LR.

In this study, we investigated the expression profiles of seed germination in APP herbicides resistant and susceptible *P.fugax* accessions at two germination time points. LS showed higher content of soluble sugar and protein than LR during germination. Accordingly, the expression of DEGs involved in carbohydrate metabolism and fatty acid metabolism had higher upregulated level in LS than in LR. Meanwhile, the transcriptome analysis identified four GA-signal-related genes with higher expression in LS and five ABA-signal-related genes with higher expression in LR during germination. Consistent with this, GA and FL promote the germination of LR plants, while ABA and PA showed greater inhibiting effect on germination of LR plants. This study provided novel insight into the gene expression profile of seed germination in *P. fugax* and identified the function genes regulating germination in herbicides resistant and susceptible accessions. It should be stressed that this study could not summarize the universal mechanism of germination delay associated with herbicide resistance mutation because the differential expression profile of seed germination was described only in two accessions. The relevant pathways or function genes in this study should be verified in other resistant plants with germination delay.

## 4. Materials and Methods

### 4.1. Plant Materials and Growth Condition

The *P. fugax* accessions were originally collected in 2012 in or nearby a grower’s field in Qingshen County, Sichuan Province, China. One of the accessions was identified to be resistant to APP herbicides, such as clodinafop-propargyl (1991-folds), fluazifop-p-butyl (364-folds), and haloxyfop-R-methyl (269-folds). The F2 seeds collected from the resistant plants (LR) had lower germination rate compared with the susceptible plants (LS) under various environment conditions [17]. The germination percentage of LR and LS were about 50% and 100%, respectively. Time to 50% germination (T_50_) of LR was 5.6 d, while that of LS was 4.7 d (unpublished data). The F2 seeds of each accession were sown in 7.5-cm-diameter plastic pots (about 20 seeds per pot) filled with sterile potting medium (mixed vegetable garden soil/cover soil, 4:1, *v*/*v*) with pH 6.3 and 13.7% organic matter. The seedlings were separately cultivated in December 2018 at approximately 15 °C/5 °C day/night temperature with natural sunlight in screen house (an 8 m by 20 m chamber framed with 2-cm iron mesh and covered overhead with a transparent plastic cover to prevent rain damage) at the China National Rice Research Institute (30.04° N, 119.55° E). The seeds were collected in Match 2019 and dried at 35 °C for seven days and stored at 4 °C under darkness for six months until use. The seed viability was tested by bromothymol blue and reached almost 100%.

### 4.2. Dynamic Changes in Germination Percentage, Soluble Sugar and Protein Content

The peeled seeds of LS and LR were surface sterilized with 70% ethanol for 1 min and 10% sodium hypochlorite for 10 min and then washed with sterile water for three times. Sterile seeds placed on the 9-cm petri dishes with one layer of filter paper that moistened with sterile water. The seeds were incubated at 20 °C/10 °C day/night cycles with a 12 h photoperiod in the growth chamber. The seed was considered as germinated when the radicle emergence to the seed length. The germinated seeds were observed by 40× microscope (Olympus SZX7, Olympus Corporation, Tokyo, Japan) at 1 d, 2 d, 3 d, 5 d, 6 d, 8 d, 9 d, and 11 d after incubated. The viability of the non-germinated seeds was tested by bromothymol blue, and the dead seeds were excluded from calculation. The germination curve was built based on the percentage of germination. Each sample had five dishes (30 seeds per dish) as replications.

Then, 0.1 g sterile seeds from each sample were collected after incubated for 0 d, 4 d, 6 d, and 8 d, immediately frozen with liquid nitrogen and stored at −80 °C. All the samples were homogenized in 1 mL cold distilled water and centrifuged at 12,000 rpm for 5 min, and then the supernatant was collected for measurement. The soluble protein content was measured by Bradford method [48]. The soluble sugar concentration was determined by the anthrone method [49]. Three replicates were analyzed for each treatment.

### 4.3. Effect of Exogenous Hormones on Seed Germination of LS and LR

The peeled seeds of LS and LR were surface sterilized and placed on the petri dishes with one layer of filter paper that moistened with 5 mL water or tested solutions, including 100 μM gibberellin (GA), 1 μM abscisic acid (ABA), 100 μM fluridone (FL), and 1 μM paclobutrazol (PA). The seeds were incubated under the same condition as mentioned above. The maximum germination (gMAX) was calculated the number of the germinated seeds divided with the total number of the viable seeds. Each treatment had five dishes (30 seeds per dish) as replications.

### 4.4. RNA Isolation and Transcriptome Sequencing

According to the dynamic change of germination rate and soluble sugar and protein content, we chose 3d and 6d as the incubated time for transcriptome analysis. The peeled seeds of LS and LR were collected for RNA extraction at 3 DAI and 6 DAI. Each sample had three biological replications named as LS_3d_1, LS_3d_2, LS_3d_3, LR_3d_1, LR_3d_2, LR_3d_3, LS_6d_1, LS_6d_2, LS_6d_3, LR_6d_1, LR_6d_2, LR_6d_3. RNA extraction and RNA-seq were performed by the Novogene Corporation (Beijing, China). RNA degradation and contamination were monitored on 1% agarose gels. RNA purity was checked using a NanoPhotometer^®^ spectrophotometer (IMPLEN, Munich, Germany). RNA integrity was assessed using ab RNA Nano 6000 Assay Kit from the Agilent Bioanalyzer 2100 system (Agilent Technologies, Carlsbad, CA, USA). A total amount of 1.5 µg RNA per sample was used as input material for the RNA sample preparations. Sequencing libraries were generated using an NEBNext^®^ Ultra™ RNA Library Prep Kit for Illumina^®^ (NEB, Ipswich, MA, USA) following the manufacturer’s recommendations.

### 4.5. Transcriptome Assembly and Functional Classification

The clean reads were generated according to the following steps: (1) the reads containing adapter were removed, (2) the reads with ploy-N were removed, and (3) the low quality reads with more than 50% of low-quality bases (Q_phred_ ≤ 20) were removed. Meanwhile, Q20, Q30, GC-content, and sequence duplication level of the clean data were calculated. All the downstream analysis were based on clean reads with high quality. All clean reads of the 12 libraries were obtained using Trinity software (v2.4.0) [50] to assemble the full-length transcript sequences. Gene function was annotated based on the following databases: NR (NCBI non-redundant protein sequences); NT (NCBI non-redundant nucleotide sequences); Pfam (protein family); KOG/COG (clusters of orthologous groups of proteins); Swiss–Prot (a manually annotated and reviewed protein sequence database); KO (KEGG ortholog database); GO (gene ontology). The transcription factors were identified by the iTAK 1.2 [51].

### 4.6. Differential Gene Expression and Enrichment Analysis

Gene expression levels were calculated using the fragments per kilobase per million fragments (FPKM) method [52]. Differential expression analysis was conducted at 6 d compared to 3 d in LS and LR using the DEGseq R package [53]. Q-value < 0.05 and|log2 (fold change)| > 1 as the threshold for significantly differential expression. All the DEGs were enriched to the GO and KEGG database by GOseq and KOBAS software [54,55]. The significant enriched GO terms and KEGG pathways were filtered by padj value < 0.05.

### 4.7. qRT-PCR Validation

The qRT-PCR was performed to verify the expression of DEGs identified from the transcriptome results. Total RNA was extracted from the LS and LR seeds under the same condition as the RNA-Seq samples using an RNAprep Pure Plant Extraction Kit (Tiangen, China). Then, reverse transcription was performed with 1 μg of each RNA sample using a cDNA Synthesis kit (TAKARA, China). qRT-PCR was performed with an iTaq Universal SYBR Green Supermix (Bio-Rad, Hercules, CA, USA) on QuantStudioTM 1 real-time PCR System and with the following thermal cycle conditions: denaturing at 95 °C for 30 s followed by 40 cycles of 95 °C for 5 s and 60 °C for 1 min. The RT-PCR primers were designed based on the coding sequences (CDS) of the tested genes (Supplemental Table S7). *UBQ* was used as the housekeeping genes. Each treatment had three biological replications, and the average values from three technical replications were used for calculating the relative expression level by 2^−ΔΔCt^ methods.

## Figures and Tables

**Table 1:** DEGs significantly up- or downregulated in LS and LR comparison settings.

Gene ID	log_2_FC (LS_6d vs. LS_3d)	log_2_FC (LR_6d vs. LR_3d)	Encoded Protein	Description
**Carbohydrate metabolism**				
Cluster-37472.6545	7.36	3.84	GPI	glucose-6-phosphate isomerase
Cluster-37472.71503	5.65	2.84	FBA	fructose-bisphosphate aldolase
Cluster-40718.0	5.96	4.88	PDHA	pyruvate dehydrogenase E1 component alpha subunit
Cluster-35171.2	6.98	5.61	PCKA	phosphoenolpyruvate carboxykinase (ATP)
Cluster-37472.39187	3.80	1.85	ADH	alcohol dehydrogenase class-P
Cluster-37472.47005	4.19	2.04	ADH	alcohol dehydrogenase class-P
Cluster-37472.85108	8.43	6.07	MDH	malate dehydrogenase
Cluster-15133.3	6.95	3.19	MDH	malate dehydrogenase
Cluster-37472.21027	9.41	4.35	CS	citrate synthase
Cluster-33485.0	6.88	5.55	OGDH	2-oxoglutarate dehydrogenase E1 component
Cluster-38121.0	7.15	5.66	LSC1	succinyl-CoA synthetase alpha subunit
Cluster-34010.0	5.12	3.97	LSC2	succinyl-CoA synthetase beta subunit
Cluster-37472.82495	9.25	6.45	SDHB	succinate dehydrogenase (ubiquinone) iron-sulfur subunit
Cluster-39676.0	7.51	4.56	SDHB	succinate dehydrogenase (ubiquinone) iron-sulfur subunit
Cluster-41938.0	7.25	4.02	PYC	pyruvate carboxylase
Cluster-37472.72264	8.37	3.93	PGD	6-phosphogluconate dehydrogenase
Cluster-37472.83492	6.66	5.71	TKT	transketolase
Cluster-37472.237	7.72	4.88	gntK	gluconokinase
**Fatty acid metabolism**				
Cluster-39490.0	7.28	4.20	ACACA	acetyl-CoA carboxylase/biotin carboxylase 1
Cluster-31226.0	7.10	4.82	FabG	3-oxoacyl-[acyl-carrier protein] reductase
Cluster-37472.4287	9.16	5.52	ACSL	long-chain acyl-CoA synthetase
Cluster-33548.0	9.20	5.34	ACAA1	acetyl-CoA acyltransferase 1
Cluster-37472.85335	9.90	5.52	ACADM	acyl-CoA dehydrogenase
Cluster-30044.0	7.77	4.75	ACAT	acetyl-CoA C-acetyltransferase
**Hormones biosynthesis and signal transduction**				
Cluster-37472.24032	9.69	3.87	KS	ent-copalyl diphosphate synthase
Cluster-37472.23672	6.42	4.78	KS	ent-kaurene synthase
Cluster-37472.70387	3.31	2.02	GA3ox	gibberellin 3-beta-dioxygenase
Cluster-51396.0	−1.69	3.01	PIF4	phytochrome-interacting factor 4
Cluster-37472.23110	−1.97	1.05	NCED	9-cis-epoxycarotenoid dioxygenase
Cluster-37472.23992	2.73	3.36	PSY	phytoene synthase
Cluster-37472.76697	1.64	2.53	LcyE	lycopene epsilon-cyclase
Cluster-37472.6593	1.32	2.78	SNRK2	serine/threonine-protein kinase SRK2
Cluster-48355.0	1.63	2.58	ABF	ABA responsive element binding factor

## Supplementary Materials

The following are available online at https://www.mdpi.com/2223-7747/9/9/1191/s1, Supplemental Table S1. Overview of the sequencing reads obtained from each sample.; Supplemental Table S2. Functional annotations of unigenes in the databases.; Supplemental Table S3. DEGs with opposite expression LS and LR.; Supplemental Table S4. Go function enrichment of up- or downregulated DEGs in LS comparison.; Supplemental Table S5. Go function enrichment of up- or downregulated DEGs in LR comparison.; Supplemental Table S6. The number of DEGs in different comparison settings.; Supplemental Table S7. The sequences of RT-PCR primers.
